# Modulation of Anti-Tumour Immune Responses by Probiotic Bacteria

**DOI:** 10.3390/vaccines8020329

**Published:** 2020-06-21

**Authors:** Georgios Aindelis, Katerina Chlichlia

**Affiliations:** Laboratory of Molecular Immunology, Department of Molecular Biology and Genetics, Democritus University of Thrace, University Campus-Dragana, 68100 Alexandroupolis, Greece; g.aindelis@gmail.com

**Keywords:** immune responses, probiotic bacteria, *Lactobacillus*, cancer, animal models, microbiota, cancer immunotherapy

## Abstract

There is a growing amount of evidence to support the beneficial role of a balanced intestinal microbiota, or distinct members thereof, in the manifestation and progression of malignant tumours, not only in the gastrointestinal tract but also in distant tissues as well. Intriguingly, bacterial species have been demonstrated to be indispensable modulatory agents of widely-used immunotherapeutic or chemotherapeutic regiments. However, the exact contribution of commensal bacteria to immunity, as well as to neoplasia formation and response to treatment, has not been fully elucidated, and most of the current knowledge acquired from animal models has yet to be translated to human subjects. Here, recent advances in understanding the interaction of gut microbes with the immune system and the modulation of protective immune responses to cancer, either naturally or in the context of widely-used treatments, are reviewed, along with the implications of these observations for future therapeutic approaches. In this regard, bacterial species capable of facilitating optimal immune responses against cancer have been surveyed. According to the findings summarized here, we suggest that strategies incorporating probiotic bacteria and/or modulation of the intestinal microbiota can be used as immune adjuvants, aiming to optimize the efficacy of cancer immunotherapies and conventional anti-tumour treatments.

## 1. Introduction

Cancer is the product of the accumulation of genetic mutations and epigenetic modifications in pathways involved in cell growth and death. The activation of genes that stimulate proliferation and survival, and the simultaneous inactivation of death-promoting genes, culminate in tumour formation and expansion [1,2]. Such modifications are the result of a combination of random mutations and environmental factors, such as diet, lifestyle, radiation, toxic substances and infectious agents [1]. The detection and destruction of malignant cells by the immune system should, in theory, be possible, at least in immune-competent individuals [3]. This process is comprised of multiple steps, proposed as the ‘cancer–immunity cycle’, and is a self-propagating procedure when initiated and allowed to expand properly [4]. Initially, tumour antigens are released from newly transformed cells, and internalized by dendritic cells (DCs) [5]. This process, when accompanied by immunogenic signals originating from dying cancer cells, neighbouring tissue undergoing stress, resident immune cells or the gut microbiota, drives forward the development of an anticancer immune response [6,7,8]. DCs then migrate and present the processed antigens to T lymphocytes on major histocompatibility complex molecules (MHCI and MHCII), promoting the priming and activation of tumour antigen-specific T cell responses [9]. The resulting effector T cells migrate to and infiltrate the tumour, where they bind and kill cancer cells, releasing additional antigens and increasing the potency of the response [10]. Unfortunately, this mechanism is not fully functional in cancer patients. Each of the aforementioned stages is susceptible to negative regulation, often arising from the tumour cells themselves, which mitigate and dampen the immune response and the effective killing of cancer cells [11].

The intestinal microbiota is a vast community of microorganisms residing in the gut. It consists mainly of bacteria, with archaea, fungi and viruses also being present [12]. The activities of the microbiota include the metabolization and uptake of nutrients; production of metabolites necessary for the host, such as vitamins and short chain fatty acids (SCFA); and competing with and excluding pathogens [13,14]. Interactions between commensal bacteria and the host are important immune regulation mechanisms, and their disturbance is implicated in a variety of disorders, including cancer [15,16]. The composition of the gut microflora has been associated with the initiation and progression of cancer, as well as the effectiveness of the treatment. These effects are mediated through the modulation of anti-tumour immune responses, and are observed in a variety of tumours, not only in the gut but also in distant tissues [17,18,19,20,21]. Disruption in the balanced interplay between microbiota and the immune system, due to alterations of the microbial composition and over-abundance of certain species, results in the establishment of chronic inflammation or local immunosuppression, and eventually neoplasia [18,22,23,24]. As a result, the microbiota can be regarded as either tumorigenic or tumour-suppressive, depending on the circumstance, and its regulation and maintenance is crucial for the overall health of the host [25,26]. The consumption of probiotics, defined as “live microorganisms that confer health benefits to hosts when administered in adequate amounts” [27], is a way of modulating the microbiota, maintaining the symbiotic relationship with the host, and introducing characteristics that ensure the optimal development of the immune system and the efficacy of cancer treatment [14,15,28].

## 2. Mechanisms of Microbiota Modulation of Anti-Tumour Immune Responses

It is generally accepted that the interaction of commensal bacteria with the immune system positively affects the development of anticancer responses through one of three proposed mechanisms. In the first, microbially-induced T cell responses cross-react with tumour antigens or provide help in the generation of anticancer immune responses. The second takes place through the microbially-induced regulation of the immune and anti-inflammatory activities by pattern recognition receptors. In the last, the secretion of metabolites modulates systemic effects [18,29]. 

As far as T cell responses are concerned, a wide range of T cell receptors are activated after interaction with bacterial structures, causing the expansion and circulation of a variety of helper T lymphocytes [30]. These T cells can then home in on tumours, perhaps due to chemokine production in the tumour microenvironment. Once there, they provide assistance to cytotoxic T cells, either through cytokine production or the induction of co-stimulatory signals [31]. Alternatively, it is possible that cross-reactivity may allow them to recognize antigens on tumour cells, as has been previously reported [32,33,34]. 

Apart from directly influencing the activation of T cells, commensal bacteria also interact with pattern recognition receptors on innate immunity cells, and thus prompt dendritic cells (DCs) exposed to them to promote potent anti-tumour Th1 and CD8+ T cell responses. Human myeloid dendritic cells treated with *Lactobacillus* strains expressed elevated levels of activation and maturation markers, such as MHCII, CD83, CD40, CD80 and CD86 on their surface. Treated DCs also secreted IL-12, IL18 and IFN-γ, but not IL-10, IL-4 or IL-13, and skewed CD4+ and CD8+ T cells to Th1 and Tc1 polarization. Th1 T cells secrete cytokines, like IFN-γ, that are mediators of various anti-tumour effects, such as cytotoxicity, angiogenesis inhibition and antigen presentation [35]. Moreover, they are directly involved in the priming of CD8+ cytotoxic T cells, which are the most efficient killers of cancer cells deployed by the immune system. Increased IL-12 production and the suppression of IL-10 was persistent even after stimulation with *Escherichia coli*-derived lipopolysaccharides (LPS) [36]. In another study, a formulation of *Lactobacillus kefiri* augmented the expression of co-stimulation and maturation markers on DCs, as well as the production of cytokines including IFN-γ and the effectiveness of CD8+ T cell activation [37]. Similar effects in the priming of human DCs towards IL-12-mediated Th1 polarization and IFN-γ production were also documented following co-culture with other lactobacilli species [38]. The combination of IL-12 and IL-18-induced production of IFN-γ was also observed in murine DCs activated by *Lactococcus lactis* subsp. *cremoris* FC [39]. Hua et al. detected the increased expression of antigen presentation and activation markers on DCs and the production of IL-12 using a mixture of three probiotics (*Bacillus mesentericus*, *Clostridium butyricum* and *Enterococcus faecalis*, Bio-Three). Furthermore, IFN-γ accumulation was observed in supernatants of CD4+ T cells and probiotic-activated DCs [40]. In summary, these observations are an indication that certain probiotic species are capable of eliciting the DC-mediated polarization of anti-tumour T lymphocytes in human cells, at least in vitro. Therefore, these and similar strains are possible candidates as natural adjuvants targeting DC immune responses.

Direct interactions between bacteria and components of the immune system are not the only mechanism of immune regulation. A plethora of microbial metabolites also exert immunomodulatory effects [41]. Microorganisms in the gut are the main source of polyamines [42,43], which have been shown to induce autophagy [44,45]. Short chain fatty acids are sensed by immune cells through G protein–coupled receptors (GPCR) [46], and butyrate has been found to modulate antigen presentation by DCs and subsequent T cell differentiation [47]. Butyrate was also reported to inhibit lymphoma tumour growth, inducing the apoptosis of cancer cells, mediated by its histone deacetylase inhibitor activity [48]. Vitamin B6 produced by gut microbes had a synergistic effect when combined with cisplatin in the treatment of non-small-cell lung cancer (NSCLC), but only in immune-competent mice displaying signs of immunogenic cell death and calreticulin exposure on the surface of NSCLC cells [49]. Moreover, histamine is involved in the regulation of DC reactions to bacterial ligands, elevating their antigen-presenting capacity [41]. 

## 3. Microbiota Composition is an Important Factor in Tumour Development and Treatment

### 3.1. Dysbiosis Impairs Immune Function and Promotes Cancer Development

As evidenced above, commensal species can have a beneficial effect on overall immunity and the generation of robust anti-tumour responses. However, the opposite is also true. When the stability of the microbiota is disrupted, in an event referred to as dysbiosis, different bacterial populations may become disproportionately represented in the gut. This imbalance can result in chronic inflammation or immunosuppression and eventually carcinogenesis [18,22,23]. 

The link between dysbiosis and colorectal cancer (CRC) is the most extensively studied. One major indication of association between microbiota alteration and cancer was the observation that the bacterial composition and diversity of tumours was significantly different than that of adjacent normal tissue [50,51,52,53,54,55]. Intriguingly, tumour-associated bacteria included strains known to impair anticancer immune responses and induce a chronic inflammatory state, such as *Fusobacterium nucleatum* [56] and *Bacteroides fragilis* [57,58], respectively. The detection of species linked with chronic inflammation is important, as this state exacerbates dysbiosis and leads to the accumulation of carcinogenic species [59,60]. In addition, epidemiological and experimental studies support the idea that reduced diversity due to the long-term use of antibiotics or diet increases the risk of CRC [61,62,63,64,65]. Interestingly, the administration of stools from CRC patients to germ-free mice and carcinogen-treated mice resulted in increased tumour formation and distinct pro-tumorigenic immune functions, as compared with stools from healthy individuals, indicating that altered fecal microbiota can promote cancer development [66]. 

However, CRC is not the only type of cancer to be associated with the composition of the intestinal microflora. Similarly to CRC, local chronic inflammation, established in response to the accumulation of specific bacterial species, appears to be the primary cause of carcinogenesis. Such cases include bile duct cancer and *Helicobacter* strains [67] or *Salmonella typhi* [68], as well as gastric cancer and *Helicobacter* pylori [69,70]. Furthermore, hepatocellular carcinoma has been associated with the metabolic activity of microorganisms. The production of secondary bile acids, such as deoxycholic acid, that are agonists of G protein–coupled bile acid receptor 1 [GPBAR1] can impede DCs function [17] and cause hepatotoxicity-induced oncogenesis [71,72]. Finally, microbiota dysbiosis has been linked to the development of breast cancer through alterations in estrogen metabolism [73,74] and the functionality of the immune system [74]. Overall, it has become evident that the maintenance of intestinal homeostasis and microbiota composition is crucial in regards to anticancer immunity.

### 3.2. A balanced Microbiota is Critical for the Effectiveness of Cancer Treatment

Taking into consideration the impact the microbiota has on immune function and carcinogenesis, it should come as no surprise that the composition and integrity of the intestinal microflora has been identified as an important factor during tumour therapy. The modulation of microbial metabolites by short term fasting or chemical agents had a beneficial effect on the tumour-inhibitory activity of chemotherapy, possibly associated with enhanced autophagy and the depletion of regulatory T cells in the tumour [45]. Sivan et al. [75] identified the significance of *Bifidobacterium* species in the development of natural anticancer immunity and the effectiveness of anti-PD-L1 administration in many different tumour models. Similarly, *Akkermansia muciniphila* has been reported to potentiate the activity of the PD-1 blockade in an IL-12-dependent manner, inducing the recruitment of CCR9+CXCR3+CD4+ T lymphocytes into tumours [76]. This dependency of PD-1 based immunotherapy on the microbiota has also been tested in clinical settings in metastatic melanoma patients [77,78]. Higher overall diversity and abundance of *Faecalibacterium* was observed in individuals responding to treatment, while lower diversity and abundance of *Bacteroidales* was detected in non-responsive patients. The underlying immune response in patients responding better to PD-1 treatment was associated with enhanced antigen presentation and T cell activity both systemically and locally in the tumour [77]. In line with these reports, the effectiveness of anti-CTL-4 therapy has also been shown to be influenced by the gut microbiota [79]. *Bacteroidales* strains were essential for the optimal immunostimulatory activity of CTL-4 blockade, and the establishment of IL-12-dependent Th1 immune responses in draining lymph nodes, as well as the maturation of intra-tumoral dendritic cells [79].

### 3.3. Disruption of the Intestinal Microflora Reduces the Efficacy of Cancer Treatment

Apart from being crucial in the generation of anti-cancer immunity, a balanced microbiota has been identified as an important mediator of cancer treatment as well. Several animal studies revealed that various anticancer therapeutic strategies were differentially effective when the commensal balance was disrupted after treatment with antibiotics. Iida et al. [80] reported that the response to both chemotherapy with the platinum salts cisplatin and oxaliplatin and immunotherapy with CpG-oligonucleotide was negatively correlated with the administration of an antibiotic cocktail (ABX). This effect was associated with reduced ROS production, and alterations in specific bacterial strains were identified as important factors in the responsiveness of mice to anticancer therapy [80]. The ABX antibiotic cocktail was also found to have detrimental effects on the efficacy of cisplatin treatment in a model of Lewis lung cancer. Mice that received antibiotics developed larger tumours, and their survival significantly decreased. The expression of VEGFA in tumours was elevated, while BAX expression was reduced. In contrast, the expression of IFN-γ, granzyme B and perforin 1 in CD8+ T cells was down-regulated [81]. Yuan et al. [82] reported that the combination of ABX antibiotic cocktail and 5-Fluoruracil (5-FU) significantly impaired the efficacy of 5-FU in mice, with the overall diversity being reduced and pathogenic species increasing at the expense of commensal bacteria. In addition, the efficacy of cyclophosphamide treatment was diminished in mice that had received anti-Gram positive antibiotics, with a reduction in Th17 responses associated with the activity of this particular therapeutic strategy [83]. Bacterial strains *Enterococcus hirae* and *Barnesiella intestinihominis* were later identified as potential candidates for the enhancement of the activity of cyclophosphamide [84]. 

In order to evaluate the effect of microbiota disruption, Derosa et al. [85] examined the outcome of anti-PD-L1 blockade treatment, in renal cell carcinoma (RCC) and NSCLC, through antibiotic administration in clinical settings. Individuals receiving antibiotics up to 30 days prior to immune checkpoint inhibitor therapy showed a decline of progress-free survival in RCC and of overall survival in NSCLC. These observations were corroborated in a study by Routy et al. [76] of patients with RCC, NSCLC and urothelial cancer. Results were consistent when all patients were considered together or according to their individual tumour. To further evaluate the hypothesis that dysbiosis was the basis of these effects, the bacterial composition of 100 patients with RCC and NSCLC before the onset of treatment was examined. Intriguingly, a correlation between better clinical outcomes and the higher richness of fecal samples was detected [76]. A similar reduction in the overall survival of cancer patients treated with CTL-4-based or PD-1-based immunotherapy, following antibiotic treatment within 30 days before therapy, was observed in another study [86]. In addition, the association of chemotherapy with antibiotic treatment was examined by Pflug et al. [87]. Both chronic lymphocytic leukemia patients treated with cyclophosphamide and patients with relapsed lymphoma treated with cisplatin achieved lower response rates and had reduced survival if they had received antibiotics. These findings suggest that an intact gut microbiota is crucial during cancer therapy, and therefore antibiotic treatment should be carefully administered in order to maximize the effectiveness of anticancer immune responses.

Given the link between dysbiosis and cancer development, an interesting question is whether the reconstitution of the microbiota is a potential approach for improvement of the anti-tumour response. Fecal microbial transfer (FMT) is a strategy considered in order to populate a patient’s colon with bacterial content from healthy individuals. FMT is currently used in the treatment of *Clostridioides difficile* infection; however, there have been reports concerning its effectiveness in other gastrointestinal disorders as well [88,89]. With regard to cancer, Sivan et al. [75] initially demonstrated that the spontaneous immune-mediated control of tumours could be transferred between responsive and unresponsive animals by FMT. Later, the same research group confirmed this effect by transferring stool material from melanoma patients, who were either responsive or not to PD-L1 treatment, into germ free mice. Animals receiving material from responsive individuals responded better and displayed slower growing tumours when implanted with B16.SIY melanoma cells [78]. The same approach of FMT from responder and non-responder patients to PD1-based treatment in germ-free mice was employed by Routy et al. [76] and Gopalakrishnan et al. [77] in epithelial cancer and melanoma, respectively. In both cases, animals transplanted with fecal microbiota from responsive patients reacted better to PD1-based treatment as well. Interestingly, similar results were observed and in the case of the anti-CTL-4 treatment of melanoma. Mice receiving material from patients reacting better to ipilimumab were themselves more responsive to treatment with the anti–CTLA-4 antibody [79]. Up to this point, the role of FMT in cancer treatment has not been elucidated, and more research is required in order to address arising issues, such as its safety and the impact of microbial biofilms on its efficacy [89,90]. However, there are ongoing studies that will hopefully help to give a better insight [20,90].

## 4. Probiotic Intervention in the Positive Modulation of Immune Responses in Gastrointestinal Cancers

Besides the preservation of the overall stability and proper interactions of the intestinal microbiota with the immune system, a different approach for the optimal development of anticancer responses is the introduction of specific bacterial strains through the diet. Various probiotic microorganisms have been extensively studied, mostly in animal tumour models. The most significant studies using probiotic microbes in cancer models are listed in Table 1. Lactic acid bacteria (LAB), including *Lactobacillus* and *Bifidobacterium* members, are among the most commonly used probiotics, and the effects of these treatments are highly strain-specific [91]. 

Naturally, the most cancer types investigated in these studies are malignancies of the gastrointestinal tract. Models of chemically-induced carcinogenesis are often employed to study the anti-tumour effects of compounds. A mixture of *Lactobacillus acidophilus, Bifidobacteria bifidum* and *Bifidobacteria infantum* (LBB), when administered to rats treated with 1,2-dimethylhydrazine dihydrochloride (DMH), resulted in reduced tumour incidence, maintaining the intestinal epithelial integrity mediated by TLR2 [92]. Similarly, in other studies, the consumption of various *Lactobacillus* species had beneficial effects in rats treated with DMH, such as the decreased number and volume of detected tumours [93,94,95], increased apoptotic cell death accompanied by modulation of cytokine production [94], and a reduced expression of COX-2 [95]. Lenoir et al. [96] reported that, in C57BL/6 mice treated with DMH and *Lactobacillus casei* BL23, the secretion of cytokines was altered in response to probiotic administration and balanced Th17 responses emerged, culminating in the reduction of tumour incidence. *L. casei* ATCC 393 also delayed the onset of cancer, reduced the number of aberrant crypt foci [97], and modified pro-inflammatory cytokine levels in serum and T cell populations in the spleen [98] in DMH-induced colon carcinogenesis in mice. In addition, in a mouse model of azoxymethane (AOM) carcinogenesis, *L. acidophilus* reduced colonic lesions by 57%, and increased serum levels of IFN-γ and IL-10, as well as the number of circulating CD4+ and CD8+ T cells [99]. Another LAB strain, *Pediococcus pentosaceus* GS4, tested in an AOM mouse model, mitigated colon cancer, inducing apoptosis in malignant colonocytes and a lower histone deacetylase (HDAC) activity [100].

Considering the known link between chronic inflammation and CRC, investigators studied the effect of probiotics in animal models of colitis-associated cancer. *L. casei* BL23 was studied in a dextran sulfate sodium (DSS)-associated mouse model of colorectal cancer by Jacouton et al. [101]. In this study, probiotic treatment had an anti-proliferative effect, detected with the decreased expression of Ki67 and up-regulation of caspase 7, caspase 9 and Bik. Moreover, immunomodulation was evident, as suggested by the down-regulation of IL-22. In addition, the effect of a commercially available mixture of LAB (VSL#3) was investigated in mouse colitis-associated adenocarcinoma. Dysplasia and tumour incidence were less prominent in probiotic-treated animals, as was the expression of proliferation markers and pro-inflammatory cytokines and COX-2, while regulatory IL-10 levels increased [102]. Furthermore, administration of VSL#3 cocktail in AOM/DSS-treated mice significantly reduced the tumour load of animals, and decreased TNF-a and IL-6 in colonic tissue [103]. Lee et al. [104] showed that inactivated nano-sized *Lactobacillus plantarum* inhibited the carcinogenesis of AOM and DSS in mice, reducing the expression of pro-inflammatory cytokines. In addition, the consumption of lactobacilli resulted in the induction of apoptosis, mediated by p53 and cell cycle arrest. Lastly, IgA secretion in the intestinal lumen was increased [104]. In another study by Mendes et al. [105] using AOM/DSS-induced carcinogenesis in mice, a mixture of *L. acidophilus*, *Lactobacillus rhamnosus* and *Bifidobacterium bifidum* led to a 40% reduction of tumour incidence, as well as lower levels of TNF-α and more IL-10 in the colon [105]. Recently, *L. acidophilus* has been found not only to attenuate weight loss caused by anti-CTL-4 antibodies but also to enhance the activity of immunotherapy [106]. This observation was associated with accumulation of CD8+ and effector memory (CD44+CD8+CD62+) T cells in the tumour. On the contrary, regulatory T cells and M2 macrophages in the tumour microenvironment were decreased. 

In a model of spontaneous carcinogenesis in C57BL/6J- Apc Min/+ mice, receiving yogurt containing microencapsulated live *Lactobacillus acidophilus,* less tumours were detected in the small intestine and the expression of proliferation markers was down-regulated. Moreover, an increased number of CD8+ lymphocytes was detected in the developing tumours [108]. In a similar study by Kahouli et al. [109], a formulation of *L. acidophilus* ATCC 314 and *L. fermentum* NCIMB 5221 significantly decreased tumour multiplicity and down-regulated the expression of β-catenin and proliferation marker Ki-67. 

**Figure 1:**
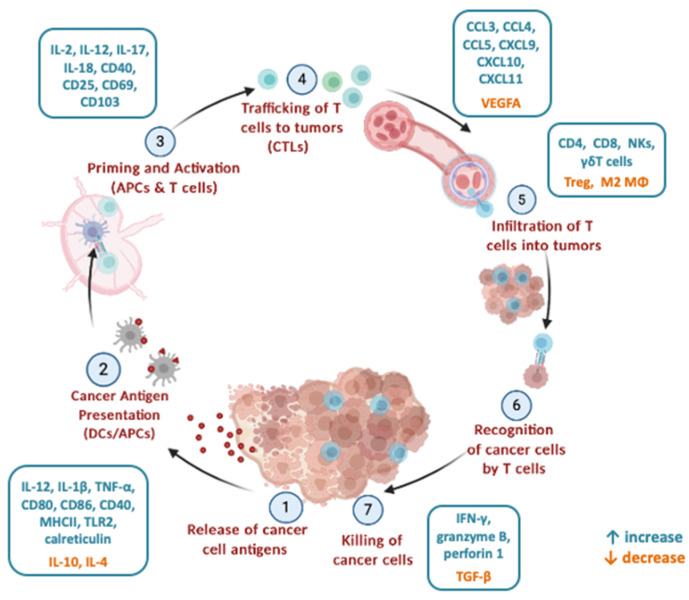
Effect of probiotic bacteria on the cancer–immunity cycle. Probiotic bacteria affect and can modulate different stages in the cancer–immunity cycle, such as cancer antigen presentation, the priming and activation of T cells, the trafficking of T cells to tumours and the infiltration of CD8+ cells into tumours, as well as the killing of cancer cells and release of tumour antigens. Several molecules are upregulated (green-blue color) or downregulated [orange color] following the administration of probiotic bacteria. The cancer–immunity cycle is adapted from [4] and was designed with Biorender (www.biorender.com).

Using a syngeneic CT26 model in BALB/c mice and *L. plantarum*, Hu et al. [111] detected a significant tumour growth inhibition, accompanied by increased IFN-γ production and CD8+ T cell and NK cell infiltration in the tumour. In a similar model, we could show that the consumption of *Lactobacillus casei* ATCC393 impaired tumour growth [112] and promoted Th1 immune responses, with an apparent increase in IFN-γ and IL-12 systemically, and in tumour infiltrating CD8+ locally, on account of the accumulation of chemokines, and in particular the ligands of receptors CCR5 (CCL3, CCL4, CCL5) and CXCR3 (CXCL9, CXCL10, CXCL11) in the tumour tissue. These chemokines play an important role in the trafficking and infiltration of T cells to tumours. Cytotoxic activity in tumours through IFN-γ and granzyme B, as well as cancer cell apoptosis, were also enhanced following oral administration of *L. casei* [113]. These studies show that probiotic bacteria affect and modulate different stages in the cancer–immunity cycle (Figure 1).

As shown above, the administration of probiotics had beneficial effects in various preclinical models of CRC. However, there is no guarantee that these observations can be transferred directly to human patients. In recent years, a number of clinical trials have been performed in order to examine the effectiveness of dietary supplementation with probiotics in CRC patients. In a double blind, randomized controlled trial, patients treated with XELOX chemotherapy that had received a mixture of probiotics (*L. casei*, *L. acidophilus*, *L. lactis*, *B. bifidum*, *B. longum* and *B. infantis*) showed milder symptoms of chemotherapy-associated inflammation. In addition, their levels of IL-6 were significantly reduced in comparison to control individuals [120]. Ishikawa et al. [121] investigated the formation of new tumours in CRC patients that had undergone tumour removal surgery. The consumption of *L. casei* resulted in a significantly reduced occurrence rate of tumours with a grade of moderate atypia or higher. Gao et al. [122] studied the microbiota of CRC patients treated with probiotics following colorectomy. Interestingly, the diversity of mucosal microbiota of treated individuals increased and, more importantly, there was a decrease in *Fusobacterium* species, which are often associated with tumours, and are thought of as tumorigenic. Notably, a similar reduction of *Fusobacterium* and a higher diversity in response to probiotic administration was observed by Hibberd et al. [123]. These effects were accompanied by the abundance of butyrate-producing bacteria, a metabolite with anticancer properties, and lower levels of *Peptostreptococcus* strains, which are also considered tumour-promoting. Finally, reports have implicated intervention with probiotics in improved immune function in CRC patients. In a double-blind, randomized controlled trial by Gianotti et al. [124], the administration of *B. longum* and *L. johnsonii* resulted in the elevated expression of T helper (CD4) and cytotoxic T (CD8) cell markers. Furthermore, both naïve and memory lymphocyte populations were increased. Likewise, supplementation with a formulation of *L. rhamnosus GG*, *B. lactis Bb12* and oligofructose-enriched inulin culminated in decreased tumour proliferation and modulation of IFN-γ and IL-2 expression in favour of the former [125]. These results are indicators that the potent probiotic-mediated anti-tumour protective effects observed in pre-clinical models have the potential to be translated to humans.

## 5. Beneficial Modulation of Anti-Tumour Immune Responses in Extraintestinal Cancers

Although cancers of the gastrointestinal tract are most commonly studied in conjunction with probiotic administration, the identification of microorganisms with immunomodulatory properties prompted investigators to broaden their research in extraintestinal cancers as well. Breast cancer is probably the most extensively studied in this regard. A report by Lakritz et al. [110] highlighted the protective role of *Lactobacillus reuteri* ATCC-PTA-6475 in two different mouse models of mammary cancer, mediated by the accumulation of regulatory T cells and the inhibition of inflammatory diseases and early stage malignant transformation. Kassayová et al. [107] evaluated the effect of *Lactobacillus plantarum* LS/07 in N-nitroso-N-methylurea (NMU) mammary carcinogenesis in rats. Despite there being no reduction in tumour growth, a decreased ratio of high-/low-grade carcinoma was detected, accompanied by reduced expression of proliferation marker Ki67. In addition, CD4+ and CD8+ T cell tumour infiltration was enhanced. In two studies by the same group, *Lactobacillus plantarum* and *Lactobacillus brevis* enriched with selenium nanoparticles induced significant tumour growth inhibition in mice injected with 4T1 cells [114,115]. In both cases, IFN-γ production and NK cytotoxicity were elevated. *L. plantarum* administration also promoted increased IL-2 and TNF-a [114], while *L. brevis* resulted in the up-regulation of IL-17 and the diminution of liver metastasis [115]. Intriguingly, milk fermented by *Lactobacillus casei* CRL 431 also retarded tumour growth in the same model of breast cancer, and serum levels of IL-6 were reduced, whereas the ratio of CD8+/CD4+ T lymphocytes was increased [116]. Dietary supplementation with *Lactobacillus acidophilus* in animals injected with 4T1 cells decreased tumour volume and also promoted IFN-γ secretion in cultured spleen cells stimulated with tumour antigens, while it severely diminished TGF-β production [117].

However, breast tumours are not the only extraintestinal cancers positively associated with probiotic consumption. As mentioned before, Sivan et al. [75] identified *Bifidobacterium* species as being indispensable for the generation of optimal immune responses against melanoma and bladder tumours. In order to confirm their hypothesis, investigators administered a mixture of *Bifidobacterium* species to mice lacking them, and the improvement in tumour control was at the same degree as that of anti-PD-L1 treatment, and more impressively the combination of both nearly abolished tumour overgrowth [75]. Oral administration of *Enterococcus hirae* 13144 enhanced cyclophosphamide-induced effects against sarcoma and increased the intratumoural ratio of cytotoxic cells to Tregs, mediated by Th1 immune responses [84]. In the same study, the administration of *Barnesiella intestinihominis* promoted the infiltration of IFN-γ-producing γδ-T cells in the tumour [84]. Furthermore, simultaneous treatment with cisplatin and *Lactobacillus* bacteria up-regulated IFN-γ, granzyme B and perforin-1 expression in a Lewis lung cancer mouse model [81]. A different probiotic cocktail (Prohep) was used by Li et al. [118] in subcutaneous hepatocellular carcinoma in mice. As a result, tumour growth was suppressed and microvessel density was lowered in an IL-17 dependent manner in treated animals. Finally, the oral administration of heat-inactivated *Lactobacillus plantarum* BF-LP284 in mice injected with Meth A tumour cells culminated in tumour volume reduction and the modulation of cytokine production and lymphocyte populations [119].

## 6. Conclusions

As evidenced above, several studies have highlighted the effect of the intestinal microflora and the consumption of probiotics in the development and effectiveness of anticancer immune responses. Commensal microorganisms emerge as a viable approach to modulating an individual’s capability to combat cancer, either in the early stages of carcinogenesis or during conventional therapies [126]. The regulation of immune function by the gut microbiota and the immunomodulatory activity of probiotic species are intriguing observations. The systemic nature of these activities implies that they can influence not only the gastrointestinal tract but also distant tissues and malignancies [18,127]. Notably, probiotic microorganisms affect and modulate different stages of the cancer–immunity cycle (Figure 1). It is important to understand that, as the majority of studies have been conducted in animal models, translation to humans is a challenge. Obviously, not all bacteria identified in animal models are part of the human microbiota, or even capable of coexistence with human hosts. The identification of those strains common in both habitats, or at least functional equivalents, will allow the narrowing of potential targets. However, taking into consideration results from clinical trials, either completed with promising results [120,121,122,123,124,125] or still on-going [20,90], we can envisage an alternative strategy worth of consideration in the treatment of cancer, based on the combination of probiotic formulations/functional foods as immune adjuvants in immunotherapies and traditional therapies.

## Figures and Tables

**Table 1:** Effect of probiotic strains in anticancer immune responses in experimental models in vivo.

Probiotic Microorganism	Experimental In Vivo Model	Effects	Ref.
*Lactobacillus acidophilus*, *Bifidobacteria bifidum* and *Bifidobacteria infantum*	DMH-induced carcinogenesis in SD rats	Reduced tumour incidence and volume, improvement of gut epithelial integrity mediated by TLR2	[92]
*Lactobacillus rhamnosus* GG CGMCC 1.2134		Reduced tumour incidence, multiplicity and volume. Decreased inflammatory cytokines. Induction of apoptosis	[94]
*Lactobacillus plantarum* AdF10 and *Lactobacillus rhamnosus* GG		Reduced tumour incidence, multiplicity and volume. Decreased expression of cyclooxygenase 2 (COX2)	[95]
*Lactobacillus salivarius Ren*	DMH-induced carcinogenesis in F344 rats	Reduced tumour incidence	[93]
*Lactobacillus plantarum* LS/07	NMU-induced carcinogenesis in SD rats	Decreased cell proliferation markers. Increased CD4+ and CD8+ cell tumour infiltration	[107]
*Lactobacillus casei* BL23	DMH-induced carcinogenesis in C57BL/6 mice	Reduced tumour incidence. Balanced Th17 immune responses	[96]
*Lactobacillus casei* ATCC 393	DMH-induced carcinogenesis in BALB/c mice	Reduced aberrant crypt foci. Decreased pro-inflammatory cytokines. Increased Treg cells in spleen	[97,98]
*Pediococcus pentosaceus* GS4	AOM-induced carcinogenesis in Swiss albino mice	Reduced cancer progression. Induction of apoptosis	[100]
*Lactobacillus acidophilus*	AOM-induced carcinogenesis in BALB/c mice	Increased serum IFN-γ, CD4+ and CD8+ cells	[99]
VSL#3	DSS-induced carcinogenesis in C57BL6 mice	Reduced tumour incidence. Decreased pro-inflammatory cytokines	[102]
*Lactobacillus casei* BL23	AOM/DSS-induced carcinogenesis in C57BL6 mice	Decreased cell proliferation markers. Induction of apoptosis. Down-regulation of IL-22	[101]
VSL#3		Reduced tumour incidence. Decreased pro-inflammatory cytokines	[103]
*Lactobacillus acidophilus*, *Lactobacillus rhamnosus* and *Bifidobacterium bifidum*		Reduced tumour incidence and volume. Decreased pro-inflammatory cytokines	[105]
*Lactobacillus plantarum*	AOM/DSS-induced carcinogenesis in BALB/c mice	Reduced carcinogenesis. Induction of apoptosis. Decreased pro-inflammatory cytokines	[104]
*Lactobacillus acidophilus* ATCC 33198		Enhanced effects of anti-CTL-4 antibodies. Increased CD8+ cell tumour infiltration. Decreased Treg cell and M2 macrophage tumour infiltration	[106]
*Lactobacillus acidophilus*	C57BL/6J Apc Min/+ mice	Reduced tumour incidence. Decreased cell proliferation markers. Increased CD8+ cell tumour infiltration	[108]
*Lactobacillus acidophilus* ATCC 314 and *Lactobacillus fermentum* NCIMB 5221		Reduced tumour multiplicity. Decreased proliferation markers	[109]
*Lactobacillus reuteri* ATCC-PTA-6475	CD-1 mice and MMTV-neu (HER2) FVB strain mice	Increased Treg cells	[110]
*Lactobacillus plantarum*	CT26 syngeneic tumour model in BALB/c mice	Reduced tumour volume. Increased CD8+ and NK cells tumour infiltration. Increased IFN-γ production. Th1 CD4+ cell differentiation	[111]
*Lactobacillus casei* ATCC 393		Reduced tumour volume. Increased CD8+ cell tumour infiltration. Increased IFN-γ and chemokine production. Th1 CD4+ cell differentiation	[112,113]
*Lactobacillus plantarum* ATCC 8014	4T1 syngeneic mammary carcinoma model in BALB/c mice	Reduced tumour volume. Increased IFN-γ, TNF-α, IL-2 production. Increased NK cell activity	[114]
*Lactobacillus brevis*		Reduced tumour volume. Increased IFN-γ, IL-17 production. Increased NK cell activity	[115]
*Lactobacillus casei* CRL 431		Reduced tumour volume. Increased ratio of CD8+ to CD4+ cells. Decreased IL-6 production	[116]
*Lactobacillus acidophilus*		Reduced tumour volume. Increased IFN-γ and decreased TGF-β production	[117]
Cocktail of *Bifidobacterium*	MB49 or B16.SIY experimental model in C57BL6 mice	Reduced tumour volume. Increased DC function and CD8+ cell tumour infiltration	[75]
*Enterococcus hirae* 13144	MCA205 fibrosarcoma model in C57BL/6J mice treated with cyclophosphamide	Increased intratumoural CD8+/Treg ratio	[84]
*Barnesiella intestinihominis*		Increased IFN-γ-producing γδ-T cell tumour infiltration	[84]
*Lactobacillus acidophilus*	Lewis lung cancer model in C57BL/6J mice treated with cisplatin	Reduced tumour volume. Increased IFN-γ, granzyme B, perforin 1 production	[81]
*Lactobacillus rhamnosus* GG, *Escherichia coli* Nissle 1917, VSL#3	Hepa1-6 experimental model in C57BL6/N mice	Reduced tumour volume. Decreased Th17 CD4+ cell tumour infiltration	[118]
*Lactobacillus plantarum* BF-LP284	Meth-A sarcoma model in BALB/c mice	Reduced tumour volume. Increased TNF-α and IFN-γ production.	[119]
